# Maize Lethal Necrosis disease: review of molecular and genetic resistance mechanisms, socio-economic impacts, and mitigation strategies in sub-Saharan Africa

**DOI:** 10.1186/s12870-022-03932-y

**Published:** 2022-11-23

**Authors:** Akshaya Kumar Biswal, Amos Emitati Alakonya, Khondokar Abdul Mottaleb, Sarah J. Hearne, Kai Sonder, Terence Luke Molnar, Alan M. Jones, Kevin Vail Pixley, Boddupalli Maruthi Prasanna

**Affiliations:** 1 International Maize and Wheat Improvement Center (CIMMYT), Km. 45, Carretera Mexico-Veracruz, El Batan, Texcoco, C.P. 56237 Mexico; 2 Stony Creek Colors, 921 Central Ave W, Springfield, TN 37172 USA; 3 Department of Biology, University of North Carolina at Chapel Hill, Chapel Hill, NC 27599 USA; 4 CIMMYT, Village Market, P. O. Box 1041, Nairobi, 00621 Kenya

**Keywords:** Maize, MLN, MCMV, SCMV, Potyvirus, Drought stress, Gene editing, QTL

## Abstract

**Background:**

Maize lethal necrosis (MLN) disease is a significant constraint for maize producers in sub-Saharan Africa (SSA). The disease decimates the maize crop, in some cases, causing total crop failure with far-reaching impacts on regional food security.

**Results:**

In this review, we analyze the impacts of MLN in Africa, finding that resource-poor farmers and consumers are the most vulnerable populations. We examine the molecular mechanism of MLN virus transmission, role of vectors and host plant resistance identifying a range of potential opportunities for genetic and phytosanitary interventions to control MLN. We discuss the likely exacerbating effects of climate change on the MLN menace and describe a sobering example of negative genetic association between tolerance to heat/drought and susceptibility to viral infection. We also review role of microRNAs in host plant response to MLN causing viruses as well as heat/drought stress that can be carefully engineered to develop resistant varieties using novel molecular techniques.

**Conclusions:**

With the dual drivers of increased crop loss due to MLN and increased demand of maize for food, the development and deployment of simple and safe technologies, like resistant cultivars developed through accelerated breeding or emerging gene editing technologies, will have substantial positive impact on livelihoods in the region. We have summarized the available genetic resources and identified a few large-effect QTLs that can be further exploited to accelerate conversion of existing farmer-preferred varieties into resistant cultivars.

## Background

Maize Lethal Necrosis (MLN) or corn lethal necrosis disease poses a severe threat to maize (*Zea mays* L.) production. Maize plants with MLN disease are often barren; the ears formed are small with no or a few deformed seeds, thereby lowering the yield drastically [1–3]. A wide range of crop losses from MLN have been reported, and a recent simulation predicted up to 73% of grain loss for susceptible varieties [4]. Discovery of germplasm carrying resistance to MLN disease has led to development and release of hybrids that are resistant/tolerant to the causal viruses. Despite considerable success of these efforts, MLN remains a threat to the food and feed requirements of resource-poor farmers of sub-Saharan Africa (SSA) given the possibility of viruses to develop new variants with potential to bypass host plant resistance.

Maize is critical for food security in Africa, providing, for example, ~ 25% of the total dietary energy intake in eastern African countries including Kenya [5, 6]. Maize kernels are consumed off the cob, roasted, cooked, and most commonly processed into a variety of products from maize flour. Because millions of small-scale farmers rely on maize to feed their families, any drop in maize yield threatens to food security and livelihoods. Other stakeholders at risk from MLN include resource-poor maize consumers, commercial seed sector, millers and transporters who engage in the maize value chain in several ways.

**Fig. 1:**
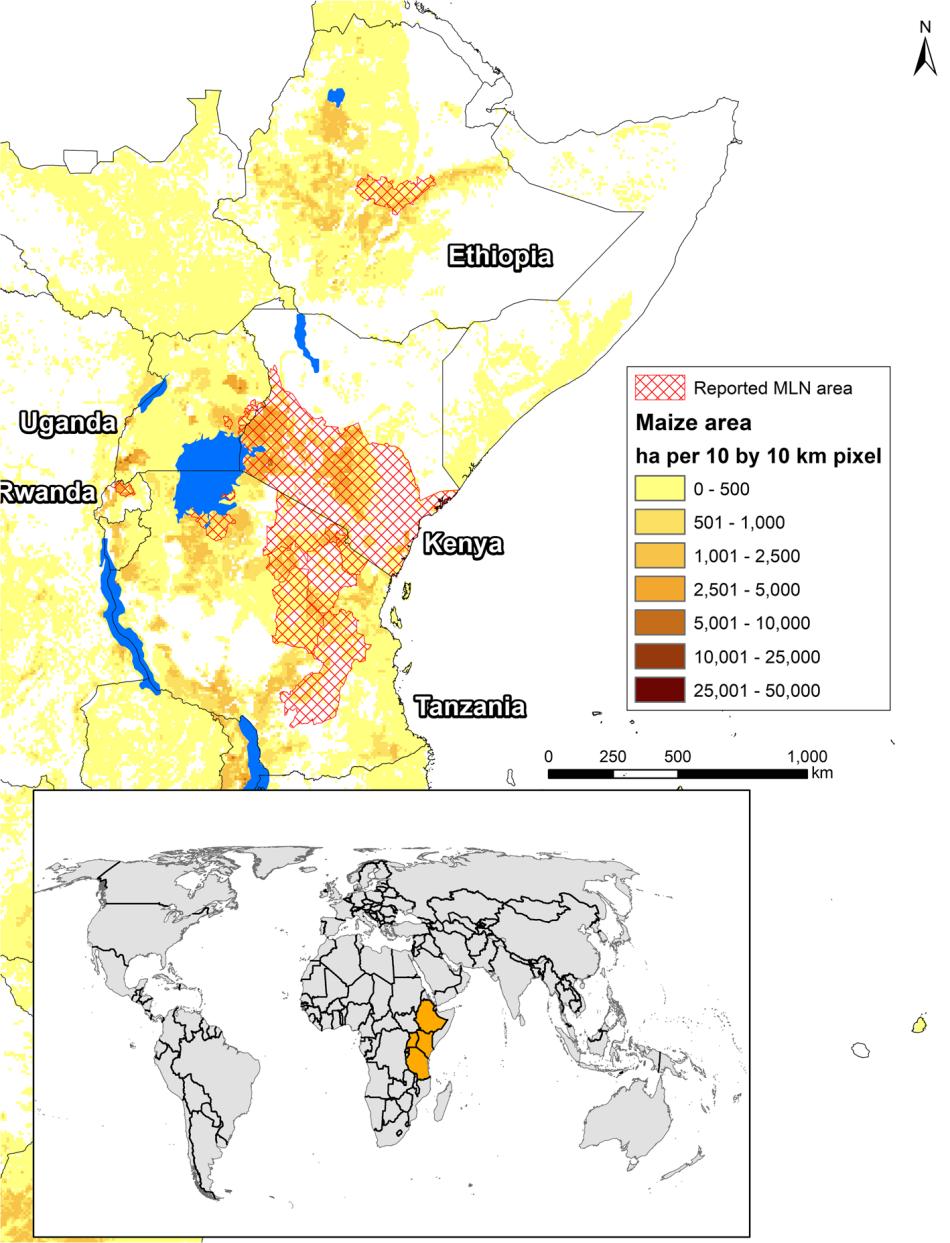
Intensity of maize cultivation and distribution of MLN disease in selected countries of Africa. Data Sources: FAO [14], SPAM 2010 [15], CIMMYT [16]

MLN was first observed in Peru in 1973 [7, 8] and later in the USA in 1976 [2, 3]. In 2011, MLN was first reported in south-western Kenya [1]. MLN-affected plots recorded yield loss worth US$ 53.2 million in 2012, $180 million in 2013 and $198 million in 2014 respectively [9, 10]. MLN disease was confirmed in six other African countries within three subsequent years (Fig. 1). Following the identification of a strong source of resistance in an inbred line from Thailand, marker-assisted backcrossing was used to convert susceptible lines to resistant and to deploy an initial set of 18 MLN resistant/tolerant hybrids in east African countries [11]. However, a recent survey in key maize seed production zones of Kenya indicated that at least 70% of seeds were infected with one or both of MLN causing viruses (MCMV and SCMV), indicating that maize production remains under threat from MLN [12]. Given the recent Russian invasion into Ukraine, the impact of production constraints including MLN are more acute and knock-on challenges to agricultural commodity markets, which may affect many countries, in particular, those were more reliant upon its agricultural exports. Among these, the hardest hit countries have been predicted to be the ones in Africa [13].

Manifestation of MLN disease is based upon a synergistic infection of a machlomovirus, maize chlorotic mottle virus (MCMV), with any one of several potyviruses, namely sugarcane mosaic virus (SCMV), maize dwarf mosaic virus (MDMV), wheat streak mosaic virus (WSMV), or Johnson grass mosaic virus (JGMV) [2, 17, 18]. Maize yellow mosaic virus (MYMV) has also been found in plants infected with MLN, but its direct role, if any, in MLN is unascertained to date [19]. MLN disease affects maize plants throughout all developmental stages [3]. Leaves of infected plants show chlorotic mottle that starts from the base of the younger leaf in the whorl and extends upwards or starts from the leaf margins progressing inward [3]. Necrosis of youngest leaf, in some cases, lead to “dead hearts” and plant death before tasseling [1]. In maturing plants, MLN causes necrosis in the tassel that progresses downwards [3]. MLN infection also causes dwarfing and premature aging of maize plants [20]. Plants resistant to one of the viruses can also develop disease symptoms due to complex interactions among other virulent viruses [21]. With predicted climate change, maize cropping will be increasingly threatened by abiotic stresses such as drought and heat stress. Most of the abiotic stress-tolerant maize lines, which have been deployed in Africa in hybrid combinations are susceptible to MLN. However, a few maize hybrids with resistance or tolerance to MLN coupled with drought tolerance have recently been deployed in east Africa [11].

In this article, we: 1) review the biological and genetic understanding of MLN disease; 2) explore factors influencing its spread and severity; 3) highlight disease mitigation options, focusing on current and potential germplasm-based contributions, including the potential of genome editing to accelerate development of novel resistant germplasm; and 4) examine the socio-economic impacts of the disease on major maize consuming countries in eastern Africa. The data driven strategies outlined in this review are immensely valuable for breeders, biotechnologists, and other stakeholders for efforts towards developing tolerant/resistant varieties to ensure food security in sub-Saharan Africa.

## Transmission of MLN pathogens

MLN has mainly been associated with co-infection of MCMV and SCMV in Africa, although one study has associated it with MCMV and JGMV [22]. A recent metagenomic analysis indicated that MCMV is the most prevalent virus associated with MLN in Kenya, while SCMV is the second [23].

### Vector transmission of MLN causing viruses

**Fig. 2:**
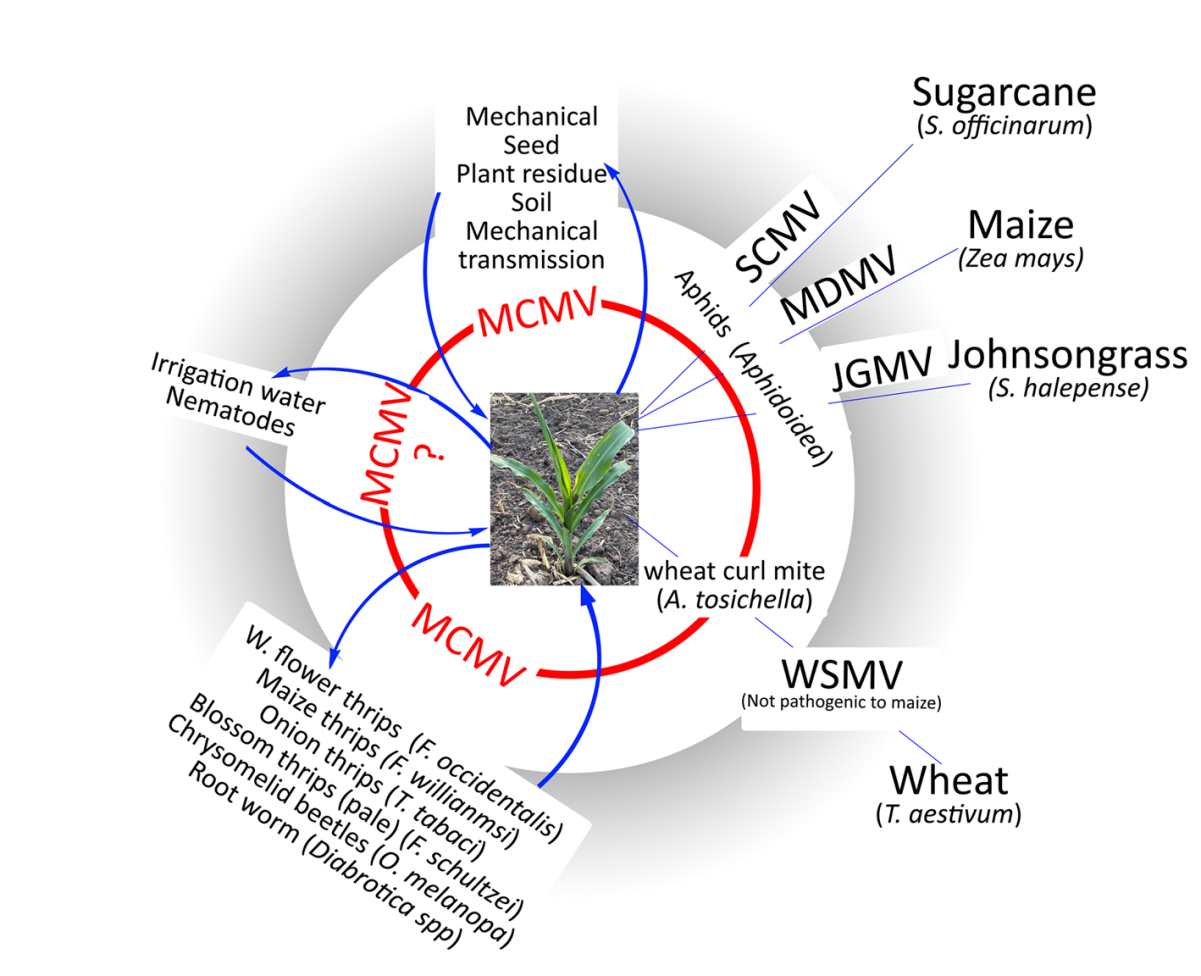
Transmission of MLN causing viruses. At the center, we have a maize plant that is amenable to infection by any of the four potyviruses on the right and MCMV on the left. The model further shows the main grass family members acting as reservoirs of the four important potyviruses (SCMV, MDMV, JGMV and WSMV) linked to MLN with aphids acting as the main transmitting vectors and a wheat curl mite transmitting only WSMV. Different dispersal mechanisms of MCMV ranging from transmission by thrips, beetles and rootworms has been presented on the left. Further, it lists mechanical, soil residues and contaminated seed as important transmission mechanisms of MCMV while the role of irrigation water and nematodes remain questionable

MCMV can be transmitted by maize thrips (*F. willianmsi)* [24, 25], western flower thrips (*F. occidentalis*) [26], beetles and root worms [27] (Fig. 2). Thrips are reported to transmit MCMV for a semipersistent period of 6 days after acquiring it [24]. MCMV is relatively new to Africa [1], while SCMV has been on the continent for a longer time, probably introduced on infected root cane (sugarcane) and maize seed [28]. SCMV and MDMV, like a majority of potyviruses, are non-persistently transmitted by several aphids that are widely distributed where maize is cultivated. It is estimated that aphids disseminate about 200 species (~ 28%) of vector-transmitted plant viruses [29] and hence control of aphids alone can significantly restrict the invasion of MLN and many other diseases into maize fields. WSMV, which is transmitted by wheat curl mite in a semipersistent manner, can lead to MLN disease symptoms in maize coinfected with MCMV. However, WSMV is not a major pathogen of maize due to genetic resistance and it has not been reported in East Africa or Asia [18, 21].

Virus transmission relies on interaction between specific receptors in the gut of the insect vector and virus coat proteins [30]. For example, potyvirus helper component-proteinase (HC-Pro) forms a bridge between the viral coat protein (CP) and certain receptors in the insect stylet to facilitate the virion retention and transmission [31]. Vectors can also be induced by acquired viruses to change their feeding behavior, resulting in higher transmission rates to selected hosts [32]. Because both MCMV and SCMV are insect-transmitted, it might be useful to understand if and how they interact with the vector system. For instance, does the feeding behavior of the insect vectors change after acquiring one or more MLN causal viruses? What happens when more than one virus is acquired by a single insect vector? Currently, there is more focus on understanding how viruses under mixed infections interact in host plants than in the vectors [33, 34]. It is important to understand interactions of mixed viral infections not just in hosts but also in transmitting vectors that also have endogenous RNA silencing mechanisms [35]. Such knowledge may suggest management approaches that manipulate the vector systems to the detriment of viruses, they transmit [31].

### Reservoirs of MLN causing viruses

Green-bridge or overlapping cropping cycles of viral host species promote the spread of MLN disease through insect vectors. Avoiding green-bridges can reduce populations of insect vectors and consequently limit the spread of the disease from one season to another. Observing a non-cropping season of 60 days for maize or any reservoir species has been shown to reduce incidence of MCMV [18]. This is important since MLN causing viruses can reside in non-maize hosts such as weeds occurring in same agroecosystem [25]. Reducing or eliminating weedy viral host species in maize fields can also reduce MLN incidence [36].

The natural host range of MCMV is restricted to the Poaceae family and includes *Sorghum bicolor, Hordeum vulgarae, Triticum aestivum, Panicum millaceu and Saccharum officinarrum* [37], although some Poaceae family members are reportedly immune [38]. Mechanical inoculation has proved that different natural and planted grass species, such as *Bromus* spp., *Digitaria sanguinalis*, *Eragrostis trichodes, Hordeum* spp., *Panicum* spp., *Setari*a spp., *Sorghum* spp. and *Triticum aestivum* can be infected by either or both MCMV and SCMV [6]. In contrast to previous studies, MCMV and SCMV were both recently detected in a dicot plant *Commelina benghalensis* belonging to family Commelinaceae, highlighting the need to screen the host range of MLN causal viruses outside the generally accepted monocot niche [39].

Several cultural practices have been proposed to aid in lowering MCMV and other MLN causing virus incidence, including non-cropping fallow periods, crop rotation with non-host plants, and sanitary practices such as use of clean equipment and getting rid of plant residues in fields [17]. Soil containing MCMV-infected crop residue has been shown to spread MCMV to subsequent cropping seasons. Further, the longevity of existence of MLN-causal viruses in the soil remains unknown. Such information could inform recommendations about effective non-cropping periods and cultural practices, discussed in this section, which would need to be balanced with other husbandry practices targeting soil fertility, organic carbon and water management. The disease may also be transmitted through infected maize seeds [40]. Diverse MCMV isolates have been detected in maize seeds [41]. Therefore, usage of virus-free seeds is important in controlling MLN in the African continent. Transmission of SCMV via seed or spread of potyviruses from infected sugarcane fields near maize fields are not considered major risks, because potyviruses responsible for MLN are endemic in maize growing areas worldwide.

## Molecular mechanism of the disease progression and resistance

As plants are sessile, most plant viruses are transmitted by insect vectors that feed on the host plant and establish an entry point by creating a wound. Upon entering the host, viruses employ a handful of their genes to orchestrate host cells’ machinery to copy their genetic material resulting in replication of new virus particles while at the same time circumventing the host’s defense mechanism. Some of the viral proteins mediate cell-to-cell movement of viral particles to facilitate spread of infection to different plant parts through host plants’ transport streams.

### General mechanism of viral infection

The establishment of viral infection is determined by the availability of host factors or susceptibility genes necessary for virus replication and movement. The disease severity is the resultant effect of plant defense to the causal virus(es) and viral suppression of host plant’s defense responses [42]. For example, host factors such as components of the maize brassinosteroid (BR) pathway induce the susceptibility of maize to MCMV infection [43]. Proteomic analysis has determined that disulfide isomerases like protein ZmPDIL-1 and peroxiredoxin family protein ZmPrx5 also enhance host susceptibility to MCMV [44]. Class I β-1,3-glucanase (GluI), associated with plasmodesmatal size exclusion, is known to promote plant virus movement [45]. Maize Elongin C (ZmElc) is another host factor that interacts with SCMV VPg and facilitates virus infection [46]. The *ZmElc* gene expression is upregulated post-SCMV infection. It also interacts with VPg of other members of the genus Potyvirus such as pennisetum mosaic virus (PenMV) and tobacco vein banding mosaic virus (TVBMV) indicating that ZmElc may be a common maize susceptibility factor for potyvirus infection. However, overexpression of *ZmElc* significantly suppressed accumulation of MCMV RNA. Conversely, MCMV accumulation was increased on knocking down of *ZmElc* gene expression in maize [46]. This indicates that ZmELc has contrasting effects on multiplication of SCMV and MCMV [47]. Though the MCMV titer is generally increased under double infection by SCMV and MCMV, it is unknown what happens to expression of *ZmElc* in that case and how the growth of both viruses is supported. Knock down of *ZmElc* expression under SCMV infection also decreases the expression of another maize susceptibility factor *eIF4E* that promotes infection of both MCMV and SCMV. Though SCMV VPg interacts with both ZmElc and eIF4E, no direct interaction has been detected between ZmElc and eIF4E [46].

The virus induced perturbation of maize chloroplast structure and function has been linked to ferredoxin-5 (Fd V) that interacts with SCMV HC-Pro protein [48]. Proteins of potyvirus also exploit the host machinery for their post-translational modification such as SUMOylation, which increases their virulence [49]. Since eukaryotic systems generally carry families of genes with redundant functions, mutation in one or more of these genes that are not essential for the plant survival can provide resistance to viral infection.

### Interaction between MLN causing viruses and the host plant

More than 50 viruses naturally infect maize [37]. Though many viral diseases are caused by single viruses, mixed viral infection of plants, as is the case for MLN, is also common in nature. Sometimes infection by one virus suppresses the introduction of the other. Alternatively, presence of the first may facilitate co-infection by the second, while in some instances both viruses may co-exist without affecting one another [29].

The MCMV combines with one of the members of the *Potyviridae* family to cause the catastrophic MLN disease [2, 3]. Typically, potyviruses enhance the titer of the partner virus [50]. The co-infection of MCMV and SCMV has shown higher accumulation of MCMV particles than single infection by MCMV, though SCMV titer remains similar between single and double infection [51]. The accumulation of MCMV is dependent on a potyviral silencing suppressor known as the helper-component protease (HC-Pro). Synergistic infection of MCMV and SCMV could significantly increase the accumulation of virus-derived small interfering RNAs (vsiRNAs) of MCMV that can potentially target dozens of host genes [51, 52]. This raises the question of whether a specific-structured single-stranded RNA in SCMV-like potyviruses promotes production of MCMV vsiRNAs.

Viral pathogens can influence the host-vector interaction by inducing changes to the host plant phenotype [53]. For example, cucumber mosaic virus (CMV) 2b protein promotes phloem ingestion of infected plants by aphids [54]. Zucchini yellow mosaic virus (ZYMV) can induce changes in leaf color and volatile emissions to attract more aphids to the infected plant. The recruitment of more aphids not only helps to spread ZYMV faster but it can also give a free ride to the co-infecting Watermelon mosaic virus (WMV) [55]. In fact, the HC-Pro of one potyvirus may interact with the CP of another potyvirus and could promote the transmission of the latter [56]. Though both ZYMV and WMV are potyviruses, it is not clear whether any of the maize infecting potyviruses can lead to increased recruitment of vectors. On the other hand, MCMV infection leads to strong induction of three volatile compounds in maize seedlings that can attract both sexes of maize thrips (*F. williamsi*) and male onion thrips (*T. tabaci*), which are important vectors for MCMV [57]. It is unknown whether recruitment of multiple MLN-causing viruses to the same vector occurs. However, combinations of up to four viruses: MCMV, SCMV, MSV and Maize yellow dwarf virus-RMV (MYDV-RMV) have been found in both symptomatic and asymptomatic maize plants that might have been transferred by one or more vectors [23]. Better understanding of co-existence mechanisms of multiple viruses and virus-virus interactions might enhance breeding efforts for developing durable-resistant germplasm.

### Genome overview of viruses associated with maize lethal necrosis

**Fig. 3:**
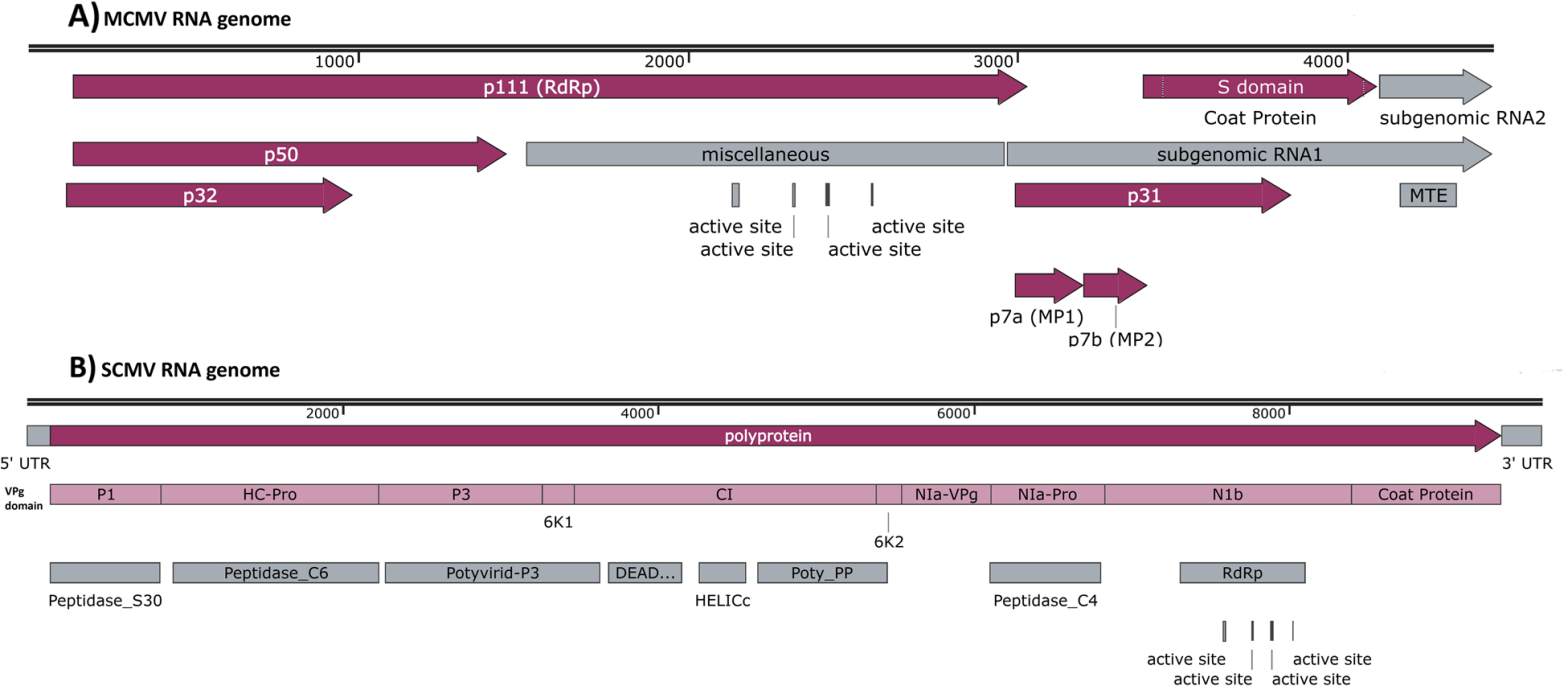
Genome map of MCMV and SCMV. **A**) The genome map of MCMV genomic RNA, isolate KS1 (NCBI Accession # NC_003627), **B**) The genome map of SCMV isolate SCMV Manyara08-TZA, complete genome (NCBI Accession # MN813967)

The MCMV virus genome comprises of a 4.4 kb single-stranded positive-sense ( +) RNA genome that potentially encodes 7 proteins (Fig. 3). MCMV produces 2 sub-genomic RNAs: RNA 1 (1.47 kb) and RNA 2 (0.34 kb) [58]. The sub-genomic RNA1 encodes four proteins P7a, P31, P7b and coat protein (CP) (Fig. 3) [59]. The N-terminal region of the MCMV CP is rich in basic amino acids. A confocal microscopic analysis has indicated that these basic residues are essential for the nuclear localization of the CP [60]. Further, it was shown that the MCMV CP interacts with importin-α and its nuclear import is most likely mediated by the importin-α/β pathway [60]. Therefore, it will be useful to investigate whether there is any allelic difference between the importins of resistant and susceptible cultivars.

Mutagenesis analysis has shown that MCMV coat protein as well as two movement proteins (P7a and P7b) are required for cell-to-cell movement in maize [61]. The P31 protein, which is a readthrough extension of P7a, is required for efficient systemic infection [61]. The P31 protein of beet necrotic yellow vein virus (BNYVV) has been shown to up-regulate pathogenesis-related (PR) protein 10 in *Nicotiana benthamiana* [62]. The BNYVV P31 is also involved in efficient vector transmission, and induction of severe symptoms in some plants [63]. MCMV P31 plays an important role in viral accumulation and symptom development by reducing the expression of salicylic acid (SA)-responsive PR genes in maize [64]. It can also bind to and enhance enzyme activity ZmPAO1 (polyamine oxidase 1) to counteract Zma-miR167-mediated defense response of the host plant [65]. Another unique protein, P32, encoded in the 5’ end of the viral genome, is required for increased accumulation of viral particles and severity of viral symptoms in maize plants [61].

The MCMV RNA also contains a cap-independent translation element (CITE) in its 3’-untranslated region (UTR). Eukaryotic translation initiation factor 4E (eIF4E) binds to MCMV CITE (MTE) despite the absence of a m7GpppN cap structure, which is generally required for eIF4E to bind to P32 RNA [66]. Therefore, eIF4E has been predicted as a soft target for engineering MCMV resistance [67]. Disruption of the function of the recessive *eIF4E* gene has resulted in resistance to potyviruses ZYMV and papaya ring spot mosaic virus-W as well as cucumber vein yellowing virus in cucumber [68]. Though no report has been made on eIF4E as a susceptibility factor for MLN disease, it will be worthwhile to determine whether allelic differences exist between susceptible and resistant cultivars of maize.

Though heat stress is generally thought to suppress the plant immunity, plants use heat shock proteins (HSPs) as common mediators for both biotic and abiotic stresses [69]. HSPs modulate the plant immunity by changing the level of accumulation and stability of PR-proteins [70]. HSP24 is involved in the resistance to a fungal pathogen in postharvest grapes [71]. Tomato yellow leaf curl virus (TYLCV) CP interaction with the HSP70 is required for viral infection [72]. Since maize HSP70 also gets upregulated during MCMV infection [44], it will be useful to verify if MCMV CP interacts with maize HSP70 and if the later regulates the MCMV infection.

The MLN-causing potyviruses have a single-stranded positive-sense ~ 10 kb RNA genome (Fig. 3). Sequence similarity among SCMV isolates from different parts of the world ranges from 79 to 90% [73]. The potyviral genome contains fixed hypervariable areas that are involved in wide-range of host adaptation [74]. Metagenomic analysis indicated that the SCMV population in Kenya is highly diverse and can be divided into three genetically distinct groups [23]. Moreover, the potyviruses of Kenya are genetically different from isolates from other parts of the world.

The potyviral genome contains a single long open reading frame (ORF) that is co- and/or post-translationally cleaved to give rise to different viral proteins. It is polyadenylated at its 3’-end and carries a viral protein genome-linked (VPg) domain at the 5’-end instead of the 5’- cap structure. The VPg is essential for viral replication [75], translation [76] and movement [77]. The interaction between VPg and eIF4E is crucial for successful viral infection [78, 79] and variations in the VPg central domain are associated with resistance-breaking in tobacco plants [80]. The m7G cap is required for RNAs to bind to the eIF4E and associate with the translation machinery. The VPg directly binds the cap-binding site of eIF4E and inhibits eIF4E-dependent host RNA export and translation in human cells [78]. Therefore, VPg can direct preferential translation of viral genome while suppressing host mRNA translation [76]. While interaction of VPg with eIF4E can promote viral RNA translation, its interaction with ZmElc may also promote viral replication [46]. The central domain of VPg also interacts with the multifunctional viral protein HC-Pro [81]. The variant of VPg protein of Tobacco Etch Virus (TEV) determines the wilting and non-wilting symptoms [82]. Human astrovirus VPg, which has sequence similarity to potyviral VPg is essential for virus infectivity [83]. The VPg domain of TEV interacts with host components to facilitate long-distance movement and systemic infection [77]. Proteomic analysis of *Arabidopsis thaliana* plants infected with TEV indicated that VPg also interacts with G-box regulating factor 6 and mitochondrial ATP synthase δ subunit [84]. Besides VPg interacts with eIF4E(iso), eIF4F and eIF4G [85]. Therefore, VPg is crucial for potyviral infection, and a detailed study is required to identify all its targets in maize. It will be useful to know whether MCMV CITE (MTE) and potyviral VPg act synergistically or compete to promote viral replication, and if the latter, which has higher affinity to eukaryotic translation initiation factors (eIF).

In general, the movement proteins are clustered at the 5’-end while the proteins related to the viral replication are skewed towards the 3’-end of the potyvirus RNA genome. A trypsin-like serine proteinase (P1) improves viral replication and cell-to-cell movement but is not required strictly for viral infectivity [86]. The helper component proteinase (HC-Pro), located right to P1, is a multitasking protein that acts as a regulator of transmission specificity by helping the virus to be held in the aphid stylets [87]. It also acts as a suppressor of host-plant resistance by suppressing post-transcriptional gene silencing (PTGS) [86, 88–90]. The central region of HC-Pro is important for the synergistic infection by PVX and PVY; however, WSMV lacking HC-Pro is competent to produce disease synergism in co-infections with MCMV [21]. Maize plants infected with either MCMV or WSMV failed to show systemic infection while plants double infected with both MCMV and WSMV (with or without HC-Pro) showed systemic infection. This indicates that a factor present in MCMV might have promoted WSMV multiplication while one or more WSMV factor other than HC-Pro led to systemic movement and infection by both viruses. Moreover, high-throughput sequencing has shown that the HC-Pro coding region of SCMV increases levels of SCMV-derived vsiRNAs in SCMV and SCMV + MCMV inoculated maize plants [51]. The P3 protein of potyviruses has been linked to symptom development, while the cytoplasmic inclusion (CI) protein is involved in RNA replication and cell-to-cell movement by forming a tunnel through the plasmodesmata [86, 91]. The coat protein participates in cell-to-cell and systemic movement in coordination with movement proteins (MPs).

### Genetic architecture of MLN-associated virus resistance in maize—knowledge from identified QTLs and genes

Host plant resistance mechanisms for MLN can be divided into two types: 1) single R-gene mediated resistance that is race-specific and 2) broad-spectrum resistance acquired due to mutation in susceptibility genes or host-factors that would otherwise support the viral invasion, systemic movement and/or viral replication. Though only a few *R*-genes conferring resistance to different potyviruses have been reported, several QTLs have been discovered showing resistance to either or both principal MLN causing viruses.

**Fig. 4:**
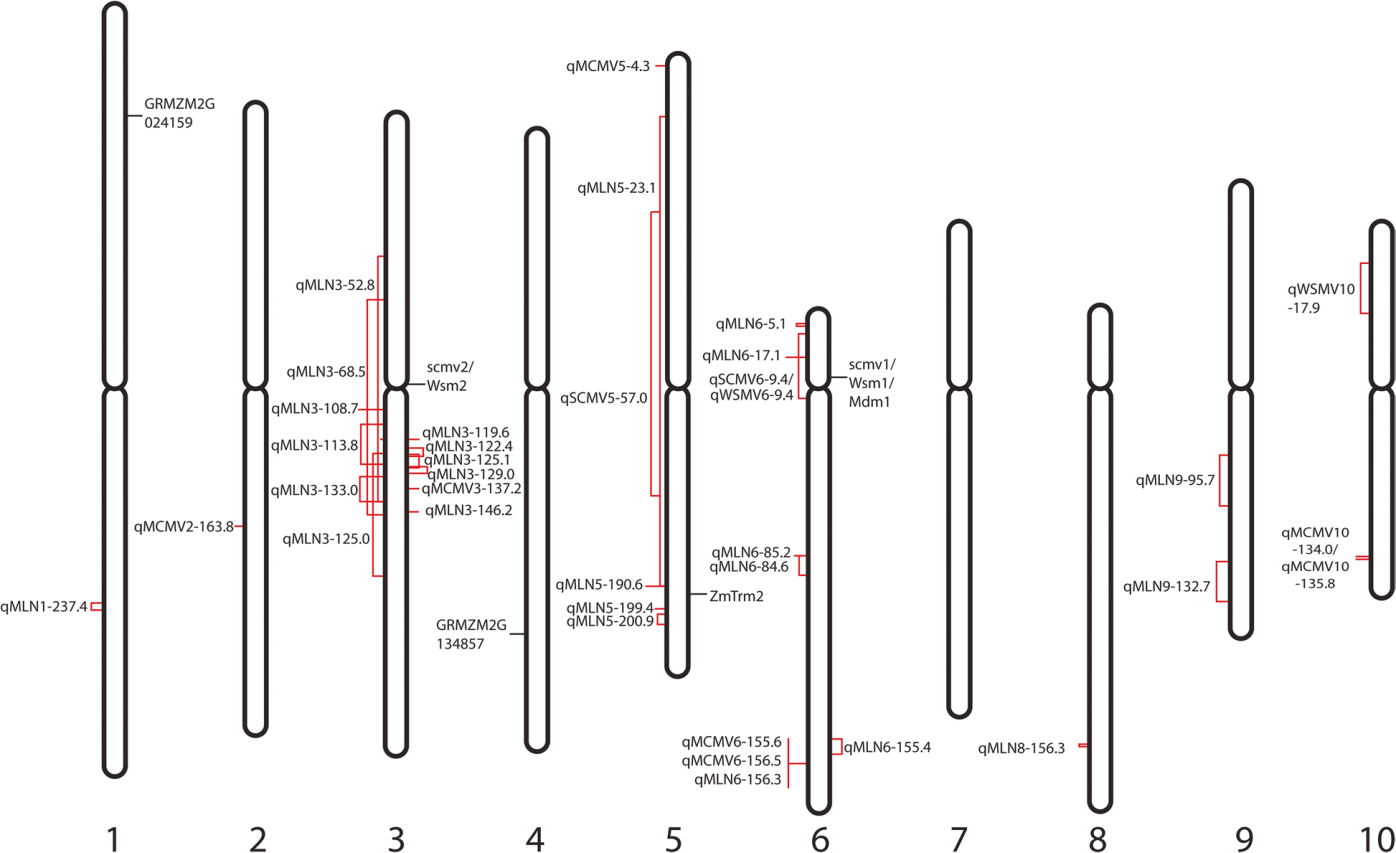
Genetic map of MLN resistance. Different QTLs with high phenotypic variance and putative genes linked to resistance to MLN or MLN causing viruses are presented. The chromosomal locations are estimated based on B73 genome V4, and may not thus represent exact location

A few maize inbred lines showing resistance to different potyviruses involved in MLN have been identified. Resistant inbred lines do not contain detectable viral titers in systemically infected leaves [92–95]. Genetic studies indicate that one- or two-gene models can explain the SCMV and MDMV resistance, while a three gene model is required to explain WSMV resistance [96]. Two major genes controlling SCMV resistance (scmv1 and scmv2) have been identified on chr. 6 and chr. 3, respectively (Fig. 4, Table 1) [97, 98]. A maize m-type thioredoxin gene *ZmTrm2* on chr. 5 also inhibits SCMV and another potyvirus, tobacco vein-banding mosaic virus (TVBMV) [99]. The *Wsm1* gene on chr. 6 provides resistance to WSMV, MDMV and SCMV [93]. Another two genes, *Wsm2* and *Wsm3*, provide resistance to WSMV. Though these two genes do not provide protection against MDMV, they function synergistically with *Wsm1* to increase resistance against MDMV. All three genes showed equal resistance against SCMV in the field. Redinbaugh et al. [92] have shown that *Mdm1*, *Scmv1*, and *Wsm1* map to same chromosomal location, while *scmv2* and *Wsm2* are co-located at another fixed locus (Fig. 4). Zambrano et. al [96] identified two QTLs on chr. 3 and 10 of maize inbred line Oh1VI that might contribute to MLN resistance by conferring tolerance to different potyviruses.

**Table 1:** Genes and QTLs for MLN resistance

Sl. No	QTL name	Author’s nomenclature	Source of favorable Allele	Viral resistance	Chr	Position/ location of closest marker(s)	LOD Score	R^2^*100/PVE %	Reference
1	qMCMV6-155.6	-	KS23-6	MCMV Resistance	6	S6_155,627,528	42.30	98.00	[94]
2	qMCMV6-156.5	-	KS23-5	MCMV Resistance	6	S6_156,591,426	39.80	94.00	[94]
3	qMCMV10-135.8	-	DR	MCMV Resistance	10	S10_135,801,262	8.90	31.00	[94]
4	qMCMV5-4.3	-	N211	MCMV Resistance	5	S5_4,322,924	9.60	38.00	[94]
5	qMCMV2-163.8	-	Oh28	MCMV Resistance	2	S2_163,825,081	10.3	18.00	[94]
6	qMCMV3-137.2	-	Oh1VI	MCMV Resistance	3	S3_137,246,834	4.3	16.00	[94]
7	qMCMV10-134.0	-	Oh1VI	MCMV Resistance	10	S10_134,058,628	8.7	11.0	[94]
8	qMLN3-108.7	qMCMV3-108/qMLN3-108	CML 550	MCMV and MLN resistance	3	108,706,910	25.34	23.73	[100]
9	qMLN6-17.1	qMCMV6-17/ qMLN6-17	CML 550	MCMV and MLN Resistance	6	17,165,743^a^	-	22.90	[100]
10	qMLN3-119.6	qMLN3-119	CML 550	MCMV and MLN Resistance	3	119,614,021^a^	-	10.90	[100]
11	qMLN3-125.0	qMLN3_130	CKDHL0221	MCMV + SCMV Resistance	3	125,077,922–169,771,952	24.64	26.00	[101]
12	qMLN3-52.8	qMLN3_142	CKDHL0221	MCMV + SCMV Resistance	3	52,804,070–142,821,031	13.19	12.71	[101]
13	qMLN3-68.5	qMLN3_142	CML543	MCMV + SCMV Resistance	3	68,596,995–146,966,676	17.68	11.09	[101]
14	qMLN3-133.0	qMLN3_142	CKDHL0089	MCMV + SCMV Resistance	3	133,048,570–142,821,031	50.02	27.46	[101]
15	qMLN5-23.1	qMLN5_190	CML494	MCMV + SCMV Resistance	5	23,135,578–191,075,472	5.14	15.91	[101]
16	qMLN5-200.9	qMLN5_202	CML494	MCMV + SCMV Resistance	5	200,938,637–204,993,639	7.62	16.65	[101]
17	qMLN6-84.6	qMLN6_85	CML 494	MCMV + SCMV Resistance	6	84,664,840–91,883,155	17.96	21.64	[101]
18	qMLN6-156.3	qMLN6_157	CKDHL0089	MCMV + SCMV Resistance	6	156,386,857–157,568,432	3.20	14.95	[101]
19	qMLN9-132.7	qMLN9_142	CML543	MCMV + SCMV Resistance	9	132,762,904–147,131,097	34.18	10.54	[101]
20	qSCMV3-57.0	-	Oh1V1	SCMV resistance	3	57,089,633–158,513,757	10.40	13.00	[96]
21	qSCMV6-9.4	-	Oh1V1	SCMV resistance	6	9,498,343–31,412,155	13.60	18.00	[96]
22	qWSMV6-9.4	-	Oh1V1	WSMV resistance	6	9,498,343–31,412,155	8.30	12.00	[96]
23	qMLN3-125.1	qMLN_03-130	CML543	MLN resistance	3	125,192,432–130,082,791	27.48	37.80	[102]
24	qMLN3-146.2	qMLN_03-146	CML543	MLN resistance	3	146,251,234–146,250,249	30.07	43.84	[102]
25	qMLN1-237.4	qMLN_01-241	CML543	MLN resistance	1	237,487,786–241,184,216	7.02	10.46	[102]
26	qMLN5-190.6	qMLN_05-190	CML444	MLN resistance	5	190,677,275–191,075,557	9.55	10.44	[102]
27	qMLN5-199.4	qMLN_05-199	CML543	MLN resistance	5	199,499,548–199,499,538	15.36	17.74	[102]
28	qMLN6-85.2	qMLN_06-85	CML543	MLN resistance	6	85,203,511–85,206,463	10.32	10.90	[102]
29	qMLN3-122.4	qMLN_03-126	CML444	MLN resistance	3	122,493,752–126,171,099	6.32	10.55	[102]
30	qMLN6-5.1	qMLN_06-06	CML539	MLN resistance	6	5,159,730 6,270,908	8.15	14.15	[102]
31	qMLN3-129.0	qMLN_03-130	CML144	MLN resistance	3	129,095,914 131,969,810	5.60	16.63	[102]
32	qMLN9-95.7	qMLN_09-100	Mo37	MLN resistance	9	95,769,540–113,201,792	5.33	16.32	[102]
33	qMLN3-113.8	qMLN_03-129	CML144	MLN resistance	3	113,820,730 129,095,914	5.58	13.97	[102]
34	qMLN8-156.3	qMLN_08-157	Mo37	MLN resistance	8	156,320,002 157,402,090	4.32	13.94	[102]
35	-	scmv1/ZmTrxh/ Zm00001d035390/wsm1/Mdm1	Pa405	SCMV resistance	6	24,034,207–24,035,363	-	-	[98, 103, 104]
36	-	scmv2/ Zm00001d041711/wsm2	Pa405	SCMV resistance	3	134,550,012–34,554,530	-	-	[97, 104]
37	-	wsm3	Pa405	SCMV resistance	10	umc163	-	-	[104]
38	-	ZmTrm2		SCMV/MDMV/WSMV resistance	5	193,879,362–193,879,998	-	-	[99]
39	-	GRMZM2G134857		MLN resistance	4	S4_199711804	-	12	[105]
40	-	GRMZM2G024159		MLN resistance	1	S1_44539940 1	-	10	[105]
41	qMLN6-155.4	qMLN06.157	KS23-5	MLN resistance		S6_155,436,477—S6_161,415,596	20.93	60.62	[106]

Since some of the QTLs had confusing nomenclature or no nomenclature assigned by authors, we generated a unique name for each QTL. In our nomenclature, the QTL starts with a code ‘q’ followed by a short code for the resistance trait. The next one or two digits before the hyphen represent the chromosome number. The numbers after the hyphen represent the chromosomal location (in Mb) of the starting coordinates of the QTL in MB with one decimal point. We have used the first decimal point without rounding the number so that it can look closer to the original QTL location. Our nomenclature is given in column 2. We have given QTL interval for most of the QTLs. Wherever the QTL interval is not available only marker position is given. AUDPC (Area under disease progression curve) values have been preferred over DS (disease severity) values wherever available

*Chr* Chromosome number, *LOD Score* Logarithm of the odds, *R*^*2*^*/PVE* Phenotypic variance explained in percentage. ‘-’: Data not available

^a^The QTL position is based on trait-associated markers of the joint linkage association mapping based on combined three double haploid populations

Similarly, several maize inbred lines have been identified with tolerance to MLN [107]. Four maize inbred lines (KS23-6, N211, DR, and Oh1VI) have been identified that develop fewer symptoms for both MCMV and MLN than susceptible controls, while having virus titers similar to those of susceptible plants [94]. This indicates that these plants are not resistant to virus multiplication though they do not exhibit the disease symptom. One or more host plant susceptibility factors promote the MCMV/MLN symptoms in susceptible plants rather than the viral genes on their own. Absence of these susceptibility factors or presence of alternate or mutant alleles lead to tolerant phenotype. Two QTLs have been identified on chr. 3 and 5 of N211, which explain more than 37% of the phenotypic variance [94]. Another unique QTL was discovered on chr. 6 of KS23-6, which alone explained nearly all of the phenotypic variance for MCMV tolerance (Fig. 4, Table 1) [94]. A similar QTL, discovered in the corresponding chromosomal region of KS23-5, explains slightly less phenotypic variance for MCMV tolerance indicating a difference in the genetic composition between both genomic loci, or an epistatic interaction with a different genomic locus. Though the smaller population size might have overestimated effects, these QTLs can be useful for breeding MLN tolerant lines and combining them can result in highly tolerant hybrids. Identification of candidate gene(s) underpinning the function these QTLs can be helpful for crop improvement via precision genetic technology. A different QTL was discovered on chr. 10 in a population derived from inbred line DR that could explain 35% of phenotypic variance [94]. Four other QTLs were discovered on chr. 1, 2, 3 and 10 of Oh1VI, which altogether explained more than 56% of phenotypic variance for MCMV resistance [94]. Two major effect QTLs, qMCMV3-108/qMLN3-108 and qMCMV6-17/qMLN6-17 were identified on chr. 3 and 6 by linkage mapping and genome-wide association studies (GWAS) conducted on three doubled-haploid populations and 380 diverse IMAS (improved maize for African soil) maize lines [100]. Each of these QTLs confer tolerance to both MCMV and MLN across genetic backgrounds and environments. Awata et al. [101] identified seven other QTLs for tolerance to MLN infections, which are stable across different genetic backgrounds and environments.

Most of the MLN tolerance QTLs are concentrated around the centromeric regions of chr. 3 or chr. 6 (Fig. 4). Multiple QTLs effecting large phenotypic variance for MLN symptoms are located on ends of chr. 6 (Table 1) and should be prioritized for additional study to identify the candidate genes and/or regulatory elements that underpin the QTL functions. Identification of QTLs at different chromosomal loci indicate that MCMV tolerance is controlled by multiple genetic elements [102] and pyramiding of these elements may provide high-level resistance/tolerance. However, many of these QTLs represent large chromosomal segments that may include multiple genes and regulating elements. Therefore, candidate gene identification will require fine mapping and functional validation, with priority placed on regions where estimated effect size is large and where recombination is more frequent (marker assisted selection within regions with higher rates of LD decay will be less effective than in low recombination centromeric blocks).

### Role of miRNA in MLN disease

The microRNAs (miRNAs) are conserved, non-coding, small RNA molecules of 20–22 nucleotides, which are known to play important roles in host plant resistance to viruses. Analysis of miRNA profiles, therefore, can help to decipher the response of the host plants to viral infection. An expression profile of maize miRNAs, obtained by challenging maize inbred line B73 with MCMV and SCMV individually and in combination revealed that expression patterns of most miRNAs were similar for single infection of SCMV and double infection with SCMV and MCMV except three miRNAs (miR159, miR393, and miR394) that were downregulated by the synergistic infection of both viruses [108]. In silico analysis indicates that these miRNAs could be playing a role in the MCMV and SCMV interaction [39]. Besides viral resistance, these miRNAs have been speculated to have roles in drought tolerance [109].  

The downregulation of miRNA159 upregulates genes associated with defense and programmed cell death such as PR genes in Tobacco though no such PR gene was upregulation was observed in miR159 expression-inhibited Arabidopsis or rice lines [110]. This indicates that there is a difference in miR159 mediated defense response between different species. The miR159 also regulates the vegetative growth and the timing of the juvenile-to-adult transition by repressing GAMYB and GAMYB-like transcription factors [111, 112]. Similarly, miR159 controls the floral organ development in coordination with miR319 [113]. Therefore, downregulation of miR159 may negatively affect the vegetative and floral development in maize plants. In Arabidopsis, miR159 is upregulated in response to ABA and drought; and silences several MYB transcription factors that are known to positively regulate ABA responses. This contrasting role of miR159 has been predicted to desensitize the effects of ABA and related stresses and allow plants to grow normally [114]. Under drought stress, miR159 also gets upregulated in subtropical maize drought-tolerant genotype HKI-1532 in comparison to the susceptible V-372 line [115]. Similarly, miR159 expression is significantly upregulated in the leaves of drought tolerant wheat lines under drought stress [116]. This suggests that the downregulation of miR159 in MLN infected plants may challenge these plants to quickly neutralize the effect of drought.

The Arabidopsis miR393 contributes to antibacterial resistance by repressing auxin signaling [117]. On the other hand, downregulation of miR393 enhanced drought tolerance by decreasing stomatal density and alleviating leaf chlorosis in barley [118]. In Arabidopsis, miR393 expression is strongly upregulated by dehydration, cold, NaCl and ABA treatments [119]. However, miR393 was significantly upregulated in response to drought stress in both tolerant maize inbred line HKI-1532 and susceptible line V-372 [115]. This indicates that the downregulation of miR393 by double infection of SCMV and MCMV may not have much impact to drought tolerance of the plant while it may compromise the disease resistance.

Overexpression of soybean miR394 in Arabidopsis leaf lowered water loss and enhanced drought tolerance [120]. Similarly, miR394 was also upregulated in drought tolerant mung bean leaves [121]. However, miR159 and miR394 were down-regulated in either MCMV only or SCMV and MCMV coinfected maize leaves [108]. This indicates that the MLN infected maize lines may show lower drought tolerance, which is important for the sub-Saharan Africa.

The expression of miR167 gets upregulated upon MCMV infection in resistant maize lines [65]. It plays a key role in antiviral resistance during single infection by MCMV only or even during double infection by MCMV and SCMV by targeting Auxin Response Factor3 (ZmARF3) and ZmARF30 in maize. In contrast, miR167 gets down-regulated under drought stress in maize seedlings [122]. It has been predicted that downregulation of miR167 might result in accumulation of its target Phospholipase D mRNAs to initiate the regulation of ABA-induced stomatal movement and antioxidant defense.

RNA silencing provides immunity in most eukaryotes against invading viruses. DICER-like (DCL) proteins play an important role in RNA silencing by recognizing and cleaving double-stranded RNA (dsRNA) from replication intermediates as well as highly structured single-stranded RNA (ssRNA) of viruses to generate small interfering RNAs (siRNAs), which, in turn, triggers specific virus clearance process in the RNA-induced silencing complex (RISC) by an Argonaute (AGO) protein [123, 124]. The DCL4 and DCL2 play an vital role in defense against certain ( +)-strand RNA viruses in a hierarchical and redundant manner [51]. The expression of DCL2 increased when plants were infected with either of the two viruses and its expression increased significantly during coinfection with SCMV and MCMV leading to a synergistic plant reaction in susceptible maize line B73 while the expression level of DCL4 was reduced [51]. In absence of DCL4, DCL2 generates 22 nt surrogate viral small interfering RNAs (vsiRNAs), which are less efficient in antiviral silencing and that may be the reason why B73 was still susceptible to SCMV and MCMV attack. Argonaute (AGO) proteins, which form core components of RNA-induced silencing complexes (RISCs), are another important class of molecule involved in RNA silencing. Two AGOs (2a and 18a) were significantly up-regulated by double infection with SCMV and MCMV. Probably they had least impact on limiting MLN viruses [51]. A further comparison of expression of AGOs in susceptible and resistant cultivars is required to understand, which AGOs play vital role in gaining MLN resistance.

Viral small interfering RNAs (vsiRNAs) can also target host genes to mediate disease symptoms in plants [52]. Both SCMV and MCMV have plethora of vsiRNAs distributed throughout their genomic RNAs, which were predicted to target genes involved in metabolic pathways, biosynthesis of secondary metabolites, transcription regulation and protein phosphorylation [51]. Double infection of MCMV + SCMV, resulted in higher accumulation of MCMV vsiRNA than single infection in maize (*Zea mays* L.) inbred line B73, which is consistent with double infection being more damaging than infection by either of the viruses alone. The siRNA expression profile of MLN infected leaves indicated that the viral capsid proteins, P7a and P7b were the most expressed genes followed by the replicase, while P32 domain showed moderate expression in the plant [39].

## Factors that may influence future MLN threat to maize

### Influence of likely climate changes

A rise in the average global temperature by 2.0–4.9 °C is likely by the end of this century [125], leading to expanded ranges and severities of many pests and pathogens [126, 127]. Although MLN is caused by co-infection of MCMV and potyvirus, infection by MCMV alone can also result in MLN like symptoms if occurring under abiotic stress [6]. MCMV infection in maize increases the abundance of HSP70 [44], which is involved in enhancing drought and heat stress tolerance [128–130]. HSP70 has been linked to increased TYLCV multiplication in tomato [72], and to virus susceptibility in Arabidopsis [131, 132] and rice [133]. The *HSP70* gene is transcriptionally up-regulated due to drought and heat stress in rice (*O. sativa*) and maize [134, 135]. Though HSP70 helps in drought and heat tolerance, HSP70 and 90 are the most frequent chaperons utilized by viruses [136]. Because HSP70 plays contrasting roles in abiotic stress tolerance and virus susceptibility, it is important to dissect the HSP70 biochemical pathway to identify unique factor(s) that help in MLN susceptibility but not heat and drought tolerance, which are critical objectives of maize breeding for sub-Saharan Africa.

Maize plants respond to increases in temperature during growth through increased respiration and faster completion of reproductive cycles. Temperatures above 32 °C during and after flowering can induce tassel blasting, pollen sterility, plant barrenness, kernel abortion or shriveled grain leading to reduced grain yield [137–141]. Farmers experiencing increased incidence of heat-stress related productivity losses may alter planting dates, irrigation frequency, cultivar use and planting locations potentially extending the green bridge and enabling more virus transmission [127].

Higher temperatures potentially favor both the insect-transmitted MLN viruses and insect vector populations [142, 143]. Elevated temperatures may negatively affect the plant responses to viral attack, especially RNA silencing, vector attractiveness, and insect probing time. It may also cause shifts in natural enemies of viral vectors, increasing transmission rates and frequency of MLN outbreaks [143–145]. Additional challenges may result from shifts in pest dynamics. Increased temperatures in the savannah may also worsen maize infestation by parasitic weeds of the *Striga* genus [146]. Viruses and parasitic plants share some resistance response pathways in plants including maize [38, 147–149]. Therefore, coinfection of viruses and *Striga spp*. might overwhelm the plant resistance mechanisms and exacerbate yield losses. Therefore, development of MLN tolerant hybrids in the background of heat and drought tolerant lines may ameliorate the problem of small-holding maize farmers of SSA.

### Influence of globalization and trade on spread of MLN

Global trade-associated movement of plant materials across borders facilitates unwanted and probably unnoticed transfer and spread of plant pathogens from endemic to non-endemic regions [126]. This may also lead to new host–pathogen, vector-pathogen or even host-vector-pathogen interactions. During 2000–2009, Kenya imported maize from at least 35 countries in the world. MLN intrusion in Kenya may be linked to maize import from the international market, although such possibility is likely impossible to confirm or refute.

Human population has been predicted to increase by 2.3 billion people by the year 2050 [150]. To meet the demand for maize by the increasing population in SSA, governments may need to import maize grains even from MCMV endemic regions [151, 152], and some of this grain may be used as seed by farmers [153, 154]. This situation risks increasing the rate of introduction of different strains of MCMV, SCMV and other non-SSA-endemic potyviruses causing MLN outbreaks.

## Current and future impacts of MLN in Africa

Although maize plays a significant role in the food security of many countries, we limit our discussion on the economic impacts of MLN to Kenya, Ethiopia, Tanzania and Uganda (Table 2).

**Table 2:** Country specific demographic information on the role of maize in food security

Country	Population (million)^a^	Projected population (2050)^b^	Poverty (%)^a^	Total domestic maize supply (MMT)	Total domestic maize supply (%) for food	Total domestic maize supply (%) for feed	Maize consumption (yearly/kg/capita)^c^	Calorie intake (kcal/daily/capita)^c^	Calorie intake from maize and (% share)^c^	Projected consumption in 2030 (yearly/kg/capita)^d^	Projected consumption in 2050 (yearly/kg/capita)^d^
Ethiopia	109.2	205.4	23.5	7.6	56	36	40.8	2285	386 (16.9)	47.1	55.5
Kenya	51.4	91.6	36.1	3.9	99	> 1	80.3	2139	709 (33.1)	66.5	54.4
Tanzania	56.3	129.4	26.4	5.5	62	22	64.5	2364	577 (24.4)	73	78.6
Uganda	42.7	89.4	21.4	2.6	74	15	49	2152	415 (19.3)	54.9	66

Sources: ^a^World Bank [155]. Ethiopia and Kenya poverty level 2015, Uganda 2016 and Tanzania 2018, ^b^World Bank [156]; ^c^FAOSAT [157]; ^d^Author’s calculation after estimating ARIMA model

Information on maize consumption and calorie intake are reported as triennium average (TE) ending 2017

### Impact of maize on food security and international trade in SSA

There is widespread heterogeneity in maize usage among major MLN affected countries in SSA (Table 2). Our analysis indicates that Ethiopia utilized 56% of its domestic maize supply as food, while almost all domestic maize was consumed as food in Kenya during 2015–2017. Uganda is the second highest consumer of maize (74%) as food, while in Tanzania 62% of maize supply contributed to food (Table 2). The contribution of maize to daily dietary energy in the sampled countries is also heterogeneous, ranging between 17 and 33% (Table 2). Irrespective of the variability in the relative importance and the uses of maize in these countries, the outbreak of MLN created havoc on the economy and food security of all. The population of these countries may grow between 78 to 129%, thereby doubling to ~ 516 million by 2050 (Table 2). We estimated the per capita maize consumption in 2050 by applying the autoregressive integrated moving average (ARIMA) model for this region. Our estimation indicates that per capita maize consumption will increase in all countries except Kenya. However, the population of Kenya is expected to double by 2050 (Table 2), which will raise the total maize consumption by more than 25%. Therefore, it is imperative to supply more maize to ensure food security in these countries.

**Fig. 5:**
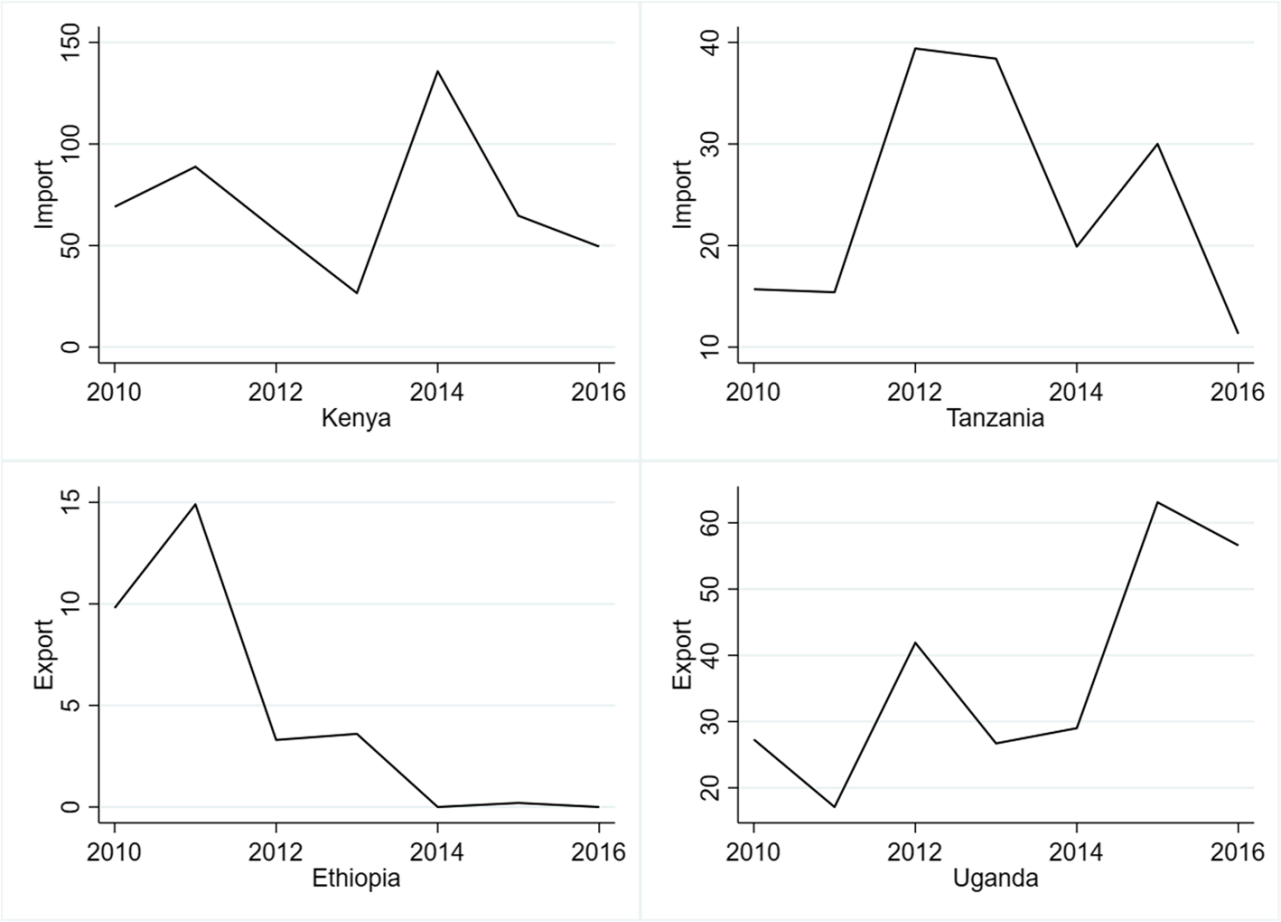
Impacts of MLN on maize imports of Kenya and Tanzania as well as exports from Ethiopia and Uganda. Source: [158]. Note: First report of MLN in Kenya was in 2011, in Ethiopia 2014; Uganda and Tanzania, 2012

Kenya is a net importer of maize. Uganda and Ethiopia are net exporters, while Tanzania is nearly self-sufficient [158]. The MLN outbreak led to a surge in maize import by Kenya in 2011 (Fig. 5). As Kenya is a major importer of Ugandan maize, the export graph of Uganda reached its peak in 2012. Similarly, the maize import of Tanzania increased more than fivefold relative to the previous year after its maize harvest was devastated by MLN in 2012 (Fig. 5). Tanzania spent US$ 24 million to support the extra maize import. The arrival of MLN to Ethiopia in 2014 drastically affected its production leading to almost shutting down of maize exports (Fig. 5). Higher demand by Kenya and Tanzania to import, and less supply by Ethiopia created an imbalance in the maize supply chain that led to increased producer price in Kenya and Ethiopia.

Kenya imports maize mainly from Uganda, Zambia, Tanzania and Mozambique [159], and is the sole importer of Ethiopian maize [159]. In addition to Kenya, Uganda exports maize to Rwanda, South Sudan, and Burundi. In the sampled countries, the poverty incidence, measured by the headcount ratio at national poverty line, is widespread ranging from 21–36% [155]. Among the major maize importers from these four countries, 38% of Rwanda’s population are below the national poverty line, while approximately 65 and 82% are below the poverty line in Burundi and South Sudan, respectively [155]. Maize trade flows of the sampled countries warn that further maize production loss due to MLN or any other causes, particularly in Uganda and Ethiopia, can have significant negative impacts on the food security in Kenya, Rwanda, South Sudan and Burundi, who rely on maize from Uganda and Ethiopia.

### Quantification of economic impact of MLN outbreak in SSA

**Fig. 6:**
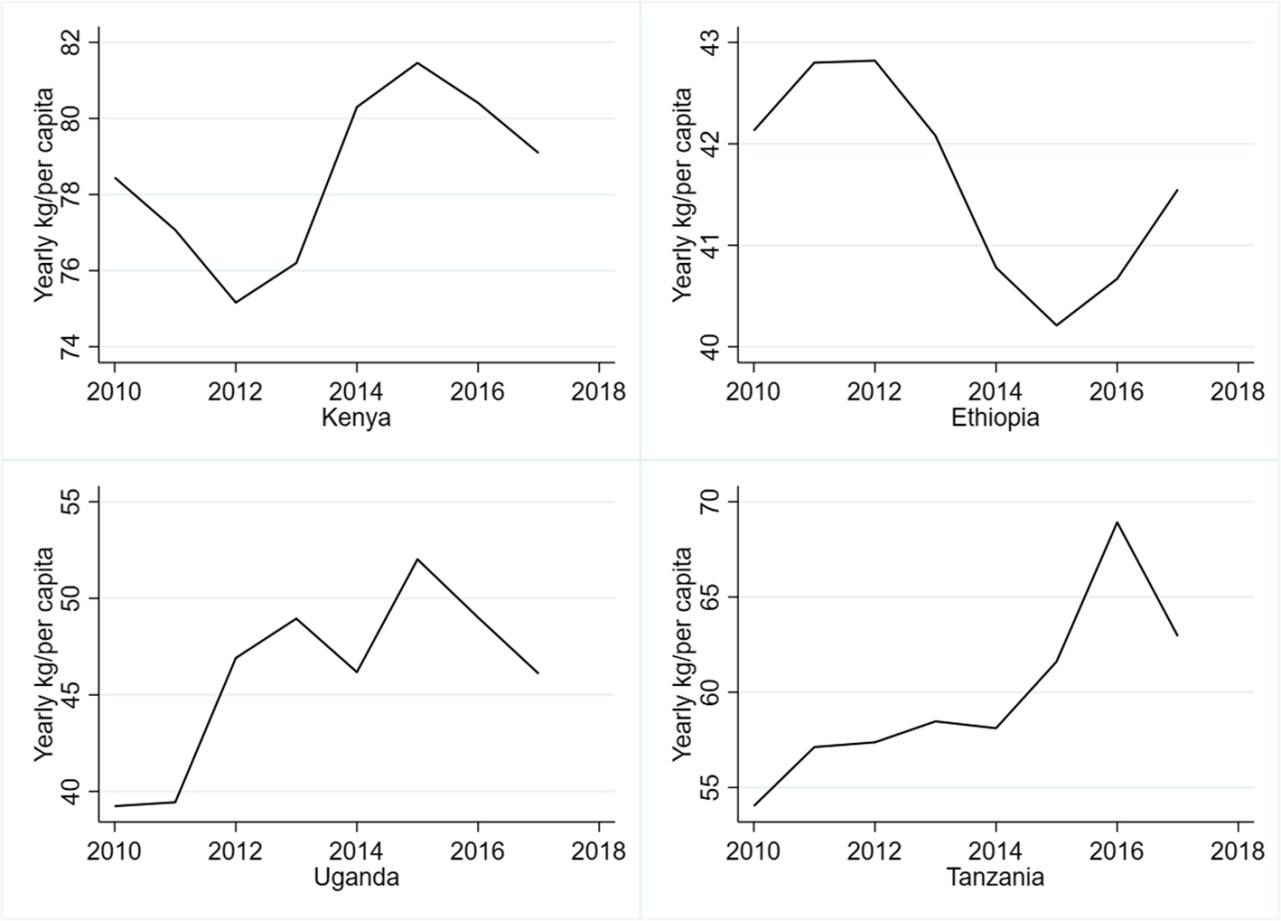
Maize consumption trends of Kenya, Ethiopia, Uganda and Tanzania (Kg/per capita/year). Source: Authors based on FAOSTAT [158]

MLN began affecting Kenya and Ethiopia in 2011 and 2014, respectively. The reduction in maize yield led to shortages that were reflected in increased producer price of maize in Kenya by ~ 30% in 2011 [160]. Similarly, the producer price of maize in Ethiopia increased by ~ US$ 16/ton in 2014 [160]. The MLN-induced maize price hikes also affected per capita maize consumption in these countries. Figure 6 shows that the yearly per capita maize consumption reached the minimum in 2012 and 2015 in Kenya and Ethiopia, respectively, compared with the previous years. The import price of maize in Rwanda also increased by 65.8% (to USD 252/ton) in 2014 compared to 2012 due to elevated maize demand [159]. MLN thus generated havoc on the already precarious food security situation in the sampled countries and their trading partners.

## Prospects of genetic engineering and genome editing in development of MLN resistant germplasm

As seen with other disease outbreaks, integrated management approaches enhance the overall response to disease outbreaks and often lead to effective lifespan of each component. Early detection of MLN pathogens is the primary requirement for managing the disease at the national and international levels. Sensitive diagnostic tools have already been developed based upon serological and molecular tools for early detection of MLN pathogens and their management. The monitoring and control of cross-border transmission of MLN viruses has partly been addressed through an MLN phytosanitary community of practice that enforces appropriate phytosanitary measures [11]. Other MLN management strategies include use of MLN tolerant and virus-free seeds, vector control, rotation and rogueing [161]. However, the economic status of the farmer and access to technologies remain the major influencer of the MLN management approaches to be adopted. Among all proposed or implemented MLN management strategies, development of MLN resistant germplasm is the most effective, durable and economically viable control measure.

Expression of viral coat protein in host plants has been demonstrated to provide resistance against target viruses [162], including to control MLN disease. Transgenic maize plants expressing MDMV strain B capsid protein did not show MLN symptoms when challenged with MCMV and different strains of MDMV [163]. Though this is a highly efficient approach for virus resistance, transgenic crops are not currently accepted in many countries [164].

CRISPR-mediated genome editing has boosted our ability to precisely target and modify sequences and expression of genetic elements. The CRISPR tools can be safely and naturally segregated through independent assortment leading to plants, free of transgenic elements [165]. This may result in higher consumer acceptance with potentially fewer regulatory hurdles than transgenic crops. Therefore, genome editing offers a novel opportunity to control viral diseases and may be exploited for development of MLN tolerant maize cultivars in countries where cultivation of gene edited crops is permitted[166]. Additionally, gene editing offers a route to broad functional validation of candidate genes potentially facilitating faster prioritization of targets to focus on in conventional marker assisted breeding schemes for regions where cultivation of gene edited crops is not yet permitted.

Large effect QTLs (qMCMV6-155.6, qMCMV6-156.5 and qMCMV10-135.8) identified in KS23-5, KS23-6 and DR-derived populations are controlled by recessive genes [94] that may be amenable to gene editing. With the recent additions of new tools like base editing [167] and prime editing [168, 169], multiple genes from both major and minor QTLs can be targeted to pyramid high-level of resistance to MLN [170].

The US Department of Agriculture (USDA) has declared that it would not regulate plants that could otherwise have been developed by conventional breeding techniques as long as they are not plant pests or developed using plant pests [166, 171]. Since a few naturally resistant maize germplasms have already been identified and such resistant allele genes can be transferred to elite lines by traditional breeding, modification of their alternative alleles to resistant version or even pyramiding of multiple such resistant loci by genome editing may not attract regulation from USDA. Similarly, plants with directed mutation in the target gene won’t be treated as transgenic (genetically modified organism, GMO) in Japan [172]. Recently, Government of India decided that genome edited products, free from exogenous DNA and falling under SDN1 or SDN2, will be exempted from biosafety assessment [173]. The China's Ministry of Agriculture and Rural Affairs has issued a guideline according to which gene-edited crop varieties will need less complicated food and environmental safety evaluations compared to true transgenics [174]. The developers need only to provide laboratory data and conduct small-scale field trials for approval by the regulatory bodies, which would take significantly less time for release than regular transgenic lines. The European Court of Justice, on the other hand, has decided to regulate genome edited plants as transgenic [175] whilst regulatory status for gene edited crops for most other countries, particularly Africa, is evolving.

## Authors’ perspective

In 2017, more than 820 million people in the world were undernourished, of whom ~ 30% were concentrated in Southern Africa [176]. More than 60% of this population suffers from moderate to extreme food insecurity. If the current trends continue, the number of people in abject poverty will increase further by 2050. It is imperative to sustainably increase agricultural productivity to eliminate hunger. Notably, as maize supplies 25% of the per capita total daily dietary energy in Africa [5], sustainable maize production is critical to food security in this region.

In general, maize yields in Africa lag far behind the rest of the world. Various biotic stresses like MLN further limit maize productivity. In 2014, about 60,000 ha of the Kenyan maize area were hit by MLN, causing approximately US$ 50 million economic loss [177]. Since more than 70% of the farm households in Africa are smallholders, development of stress-tolerant varieties and rapid scaling out of those technologies can contribute to food security and income for resource-poor farmers. If we assume that 50% of the total affected area can be planted with MLN tolerant/resistant maize, which can lower yield loss by 80%, the economic benefit can be as much as US $ 20 million every year in Kenya alone. The need to develop and disseminate MLN resistant maize cultivars in this region is apparent and imperative.

Besides MLN, maize streak virus, parasitic weeds *(Striga hermonthica* and *Striga asiatica),* ear rot fungi, maize stem borers, fall armyworm, gray leaf spot, *Bipolaris maydis* and downy mildew are economically important biotic constraints for maize production in Africa [178, 179]. Abiotic constraints such as low soil fertility, drought and heat stress also cause significant yield losses [178, 179]. Therefore, MLN tolerant varieties must be resistant to multiple other stresses. Genome editing shows promise for developing MLN resistant varieties directly in elite African maize cultivars without affecting other stress tolerances, agronomic performance, and consumer-preferred characteristics. Although it is beyond the scope of this review, it is important to implement policies, equitable seed system models, and social communication strategies to support the adoption of new technologies, including improved varieties, which is complex and seldom rapid [180].

## Conclusion

Demand for maize in SSA may increase by 140% by 2050. Poor farmers and consumers, particularly the poor in net maize importing countries, are the most vulnerable populations if maize production fails to rise to meet this demand. MLN is a serious threat to maize production in SSA, and its sustainable control will need a multipronged approach comprising vector population control, clean seed, clean soil, and most importantly host plant resistance. Genome editing is a particularly promising approach to improve host plant resistance, because it avoids disadvantages of linkage drag associated with traditional backcross breeding. Our review of the genetics determining MLN vector capability, MLN viral effectiveness, and maize host plant resistance mechanisms suggests several potentially effective targets to contribute to effective control of MLN.

## Data Availability

Not applicable.
